# Cancer antigen 125 in ovarian cancer surveillance: a decision analysis model

**DOI:** 10.3747/co.2007.144

**Published:** 2007-10

**Authors:** M.L. Hopkins, D. Coyle, T. Le, M. Fung Kee Fung, G. Wells

**Affiliations:** * Division of Gynecologic Oncology, University of Ottawa, Ottawa, Ontario; † Department of Epidemiology and Community Medicine, University of Ottawa, Ottawa, Ontario

**Keywords:** Decision analysis, CA-125, preferences

## Abstract

We used decision analysis techniques with Markov cohort modeling to examine the role of cancer antigen 125 (CA-125) in follow-up surveillance strategies among patients with advanced ovarian cancer. Utilities were derived from a societal perspective.

Using quality-adjusted life years (qalys) as the outcome variable, the value of CA-125 monitoring for asymptomatic women with ovarian cancer was found to be reduced as compared with a strategy that includes CA-125 testing. Decisions to include CA-125 in surveillance strategies for ovarian cancer patients should be made after discussion with full disclosure of the preference-sensitive nature of CA-125. The model demonstrates that preferences and perspective can influence decisions in cancer care.

## 1. INTRODUCTION

Many authors have reported on the prognostic significance of the cancer antigen (CA-125) assay in ovarian cancer patients 1–3. The assay has become established in clinical practice, and it is widely used to monitor response to chemotherapy, including chemotherapy given in the recurrent setting 4, 5. It has also been used in many academic centres to facilitate early diagnosis of relapse 6.

Although a rising CA-125 level in a patient with clinical complete remission is highly predictive of a symptomatic recurrence (median time to development of symptoms, physical signs, or radiographic evidence of recurrent disease is 3–6 months), there is no evidence that immediate treatment with salvage chemotherapy is more effective than reserving such treatment until the time that other manifestations of recurrent disease appear 7–11. Furthermore, patients have been shown to anticipate the assay results with heightened anxiety at each follow-up visit, a condition described as the “CA-125 addiction” 12.

Recurrent ovarian cancer is incurable, and second-line therapy is less effective and less tolerated than is chemotherapy used in the primary setting. Therefore, the goals of treatment for relapse include providing disease control and symptom palliation with a well-tolerated chemotherapy regimen and providing support and maintenance of good quality of life (qol). It is widely accepted that patients who have symptoms and a rising CA-125 concentration should start treatment. There is debate regarding whether asymptomatic patients with recurrence should be treated, and further, whether CA-125 monitoring should be a routine component of disease surveillance for women with ovarian cancer. It has been suggested that, until proof has been developed that early recognition and treatment of relapse improves patient outcomes, routine measurement of CA-125 for disease surveillance be abandoned 13.

The precise utility of diagnosing biochemical ovarian cancer relapse is unknown, but its impact must be defined in terms of survival, response to salvage chemotherapy, and ultimately, the patient’s QOL^14^. Because of the controversy and the multiple health and qol-related issues surrounding the use of CA-125 assays, decision analysis techniques with Markov cohort modeling can be useful in quantitatively and transparently evaluating the role of CA-125 in the follow-up management of women with ovarian cancer. Our objective was to use decision analysis techniques to examine the role of CA-125 in surveillance strategies for women, based on a societal perspective.

## 2. PATIENTS AND METHODS

### 2.1 Study Population

The population considered within the decision analysis consisted of women with stage iii or iv ovarian cancer who had attained a complete response to primary treatment for their disease. The median age of patients presenting with ovarian cancer is 63 years and the start age for the purposes of the decision analysis model was therefore set to that level 15.

### 2.2 Study Comparators

The decision for or against incorporating CA-125 monitoring into follow-up management allows for consideration of two alternative strategies:

- Intensive monitoring (that is, with CA-125)
- Non-intensive monitoring (that is, without CA-125)

Women in the intensive monitoring arm of the decision analysis were to undergo cyclic CA-125 testing if asymptomatic. All patients with symptomatic recurrence were to receive second-line chemotherapy. Because the serum CA-125 test is an imperfect diagnostic test, Bayes’ revision was applied to ensure the use of valid probabilities. The data on the sensitivity and specificity of the CA-125 test came from a systematic review completed by our group (submitted for publication), and the prevalence probability of advanced stage ovarian cancer came from *Canadian Cancer Statistics 2003* 16.

### 2.3 Markov Model

**FIGURE 1:**
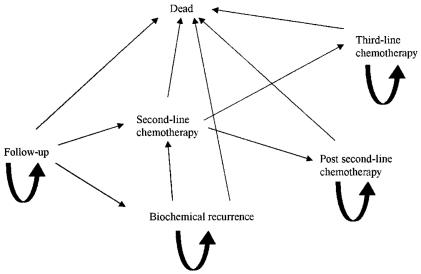
Possible options for health state occupation in the decision analysis model with Markov cohort.

Figure 1 schematically depicts the various health states through which a patient can possibly transition in the decision analysis model. The lifelong course of events for patients with stages iii and iv ovarian cancer are simulated as a Markov cohort.

At initiation of the model, all women with ovarian cancer are assumed to initially have a complete response to a combination of surgery and chemotherapy and to traverse through follow-up time until all members of the group die or reach 100 years of age. Over time, a woman can remain well, recur, or die from recurrence or competing risks. Over a series of cycles, women move from one state to another as determined by transition probabilities that can differ with time and age. Cycle length for the model is 3 months, corresponding with the initial follow-up interval after completion of primary therapy.

The average number of cycles before death is used to determine the life expectancy associated with each strategy. Time spent in each health state is weighted by an associated utility value to allow for an estimation of quality-adjusted life years (qalys).

The model was developed using Data 4.0 software (TreeAge Software, Williamstown, MA, U.S.A.) and Excel 2000 software (Microsoft, Redmond, WA, U.S.A.) written for use on an IBM personal computer.

### 2.4 Utility Assessment

Reduction or improvement in qol resulting from a diagnosis of recurrence was considered by analyzing qalys. Utility scores, which combine an individual’s preferences for a specific health state with morbidity data, are further combined with information on life expectancy to produce the single weighted measure qaly. “Utility” has been defined as the level of desirability associated with a particular outcome or health state 17. No evidence exists in the literature to describe the associated disutility of being treated with chemotherapy when asymptomatic (associated with a true positive CA-125 test or a false positive CA-125 test) or the utilities of receiving second-line chemotherapy for a first recurrence, having a complete response to second-line chemotherapy, or receiving third-line chemotherapy for a second recurrence. Given that chemotherapy for recurrent ovarian cancer may cause serious side effects and have uncertain or limited benefits, the risks of the therapy must be weighed against its value.

To make decisions regarding preferred therapy, value judgments are required. In clinical practice, this type of evaluation is currently made in an informal and implicit manner. Recent methodologic developments in shared decision-making now allow for an explicit assessment of preferences where more than one option for treatment is available. In utility approaches, a variety of scaling methods are available to assign a numeric value on a scale where 0.0 represents death and 1.0 represents perfect health. Approaches include the standard gamble, the time trade-off, and the visual analogue scale (vas) 18.

For the present study, a convenience sample of 20 women from the community were selected to derive utilities for 8 different health states relevant to the decision analysis. Respondents had no personal experience with ovarian cancer. The use of data from a community sample corresponds with recommendations from The Panel on Cost-Effectiveness in Health and Medicine, a non-federal panel convened by the U.S. Public Health Service with expertise in cost-effectiveness analysis, clinical medicine, ethics, and health outcomes measurement 19.

Health states were developed based on the attributes of the EuroQOL questionnaire 20 and were valued using the vas. The vas was organized vertically like a thermometer. The 8 different health states for the decision analysis were organized at the periphery of the scale. In this way, all health states could be considered simultaneously. The description of each health state contained information on 6 different domains of health, including mobility, self-care, main activity, social relationships, mood, and side effects of treatment.

### 2.5 Probability Estimates

Probabilities were derived from published work where possible, but in some instances, no published data were available for inclusion in the model. Consequently, expert judgment was required to estimate some probabilities. Annual recurrence rates for ovarian cancer were estimated based on 5-year survival data for patients with stages iii and iv ovarian cancer 16, 21. Table I gives the probability estimates used in the decision analysis. To allow for the possibility of a biochemical recurrence leading to death (because of surveillance without CA-125), a relative risk of mortality was added in the non-intensive monitoring arm for the purposes of Monte Carlo simulation:

$$\log_{n}(\mu =1,\sigma =0.05).$$

### 2.6 Assumptions

To keep the model as simple as possible and yet reflective of the follow-up characteristics of patients with advanced ovarian cancer, several assumptions are made.

Within the model, recurrence rates are assumed to be constant over time. Although recurrence rates are known not to be constant over time, this assumption is unlikely to affect the results of the analysis, because recurrence rates are identical between the two possible follow-up strategies.

Also, once a patient is diagnosed with a second recurrence (requiring third-line chemotherapy), no further complete response will be possible. Patients in that situation will remain on treatment until death. Although no evidence describes the frequency and duration of complete response post third-line chemotherapy, clinical experience suggests that very few patients (fewer than 10%) are likely to reach this health state. Indeed, it has recently been proposed that ovarian cancer patients who have achieved a remission following primary therapy not have follow-up visits until 2 years after completion of their first-line chemotherapy.

### 2.7 Analysis of Uncertainty

Uncertainty regarding the expected values of outcomes of interest was explored through the use of Monte Carlo simulation. Monte Carlo simulation requires the specification of probability distributions for all input parameters within the model. Repeated estimates of outcomes are obtained by re-running the model using different values for each data input, randomly selected from the probability distributions. Utility variables for the decision analysis were assigned normal distributions. Empiric probability variables were assigned a beta distribution. Variables without a known distributional form (that is, those with assumed values or those with values based on other published reports) were assigned a triangular distribution.

In addition to Monte Carlo simulation, uncertainty was further assessed using sensitivity analysis, whereby values for key parameters were changed to assess the sensitivity of results.

## 3. RESULTS

The results of the analysis are presented in terms of the expected values of outcomes based on a Monte Carlo simulation with 3000 replications. Base-case results are presented for a woman aged 63 who is diagnosed with ovarian cancer (stage iii or iv) and who has a complete response to primary treatment. Analysis demonstrates that life expectancy is essentially no different between the two strategies: the expected difference is 0.0 years [95% confidence interval (ci): 0.00 to 0.001]. However, the expected qaly value is slightly higher for non-intensive monitoring than for intensive monitoring: a difference of 0.28 years (95% ci: 0.09 to 1.05). Costs are higher for intensive monitoring as compared with non-intensive monitoring: a difference of $801.63 (95% ci: $402 to $2099). Table II provides a summary of incremental values from the Monte Carlo simulation.

**FIGURE 2:**
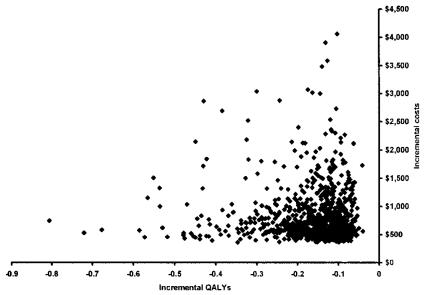
Scatter plot of incremental quality-adjusted life years (qalys) versus incremental cost of monitoring with cancer antigen 125.

Overall, non-intensive monitoring is dominant over intensive monitoring; it is both more effective (in terms of qalys) and less costly. This result is consistent across all 3000 Monte Carlo replications (Figure 2).

### 3.1 Sensitivity Analysis

We tested the robustness of the overall conclusions by performing sensitivity analyses. The two main branches of the model are identical, except for the inclusion of the CA-125 test and a possible relative risk of dying from an unrecognized biochemical relapse.

Intensive monitoring is inferior to non-intensive monitoring throughout the range of sensitivities and specificities, owing largely to the effect of preferences and values associated the health state of being diagnosed with a biochemical relapse. Because CA-125 more confidently identifies a biochemical relapse, the overall expected utility of intensive monitoring decreases, as reflected by the fact that the survey participants viewed a cancer recurrence quite negatively. As the specificity of the CA-125 test increases, the expected value of intensive monitoring is seen to increase. This result is predictable, because the ability of a test to accurately “rule out” a disease is desirable.

The sensitivity analysis comparing the two follow-up strategies was performed with variation according to the relative risk of death resulting from failure to diagnose a biochemical relapse. A “threshold” effect was seen at a relative risk value of 1.0. Indeed, intensive monitoring could be desirable if a meaningful survival advantage attached to early recognition and treatment of recurrence based on elevated CA-125 levels.

## 4. DISCUSSION

Decision analysis allows for clinical problems to be organized structurally into their component parts. We developed a decision model to describe the current options regarding the use of CA-125 in the follow-up of women with advanced ovarian cancer.

The model highlights the lack of published data on a number of vitally important statistics in ovarian cancer, such as the probabilities of dying from complications of chemotherapy and disease burden. No published information on annual recurrence rates according to disease stage are available. The absence of such data is a limitation of this decision analysis. Clearly, collection of the foregoing information is an important goal for future work and database development in ovarian cancer.

An important attribute of decision analysis is the possibility of explicitly providing a place for a patient’s point of view and values. Varying perspectives can be expected to produce variation in the quantitative results of decision analysis. For example, it was previously shown that physicians and patients do not share a common view about health-related matters, especially issues relating to pain, physical status, and psychological wellbeing 26–28. Calhoun *et al.* 29 recently published an analysis of utility assessments for various ovarian cancer treatments and demonstrated that assessments of the impact of chemotherapy-related toxicity vary depending on the perspective of the individual responding to the questions. Specifically, the study examined perceptions of the severity of chemotherapy-related toxicity among physicians, ovarian cancer patients, at-risk women, and women in the general population. Health states with toxicity were associated with less favourable assessments than were health states with no toxicity. The ovarian cancer patients and the women at risk for development of ovarian cancer viewed health states with toxicity similarly and more favourably than did women in the general population. However, patient assessments varied depending on whether they had previously experienced life with toxicity from chemotherapy. An objective of future work will be to compare the results of this decision analysis from a societal perspective with one from a patient perspective. The overall results would likely be different and reflective of a preference for CA-125 testing. Most ovarian cancer patients desire tests to assess their disease status. Information is viewed positively.

With increasing attention to shared decision-making in medicine, assessment of patient preferences will become more main stream. Physicians must understand how preference-sensitive inputs can affect health care decisions. Perspective can have a strong effect, not only for individual decisions about health care, but also for the formulation of health policy and practice guidelines. An example is found in a clinical practice guideline for menorrhagia, published by the Royal College of Obstetricians and Gynaecologists in 1998, which recommend that women’s preferences for watchful waiting, medical treatment, or surgery be considered when decisions are made about appropriate care.

The benefits of detecting biochemical ovarian cancer recurrence are uncertain. Currently, there is no evidence that recognition and treatment of biochemical recurrence has any survival benefit. The results of an ongoing European trial to address this question are awaited. In the meantime, the potential negative impact of a recurrence on qol indicates that merit accrues to the evaluation of preferences for CA-125 testing in surveillance strategies for ovarian cancer.

## Figures and Tables

**TABLE I:** Probability estimates for ovarian cancer follow-up per 3-month Markov cycle

Variable	Reference	Variable name in model	Estimate (range)	Comments
Probability of death due to competing risks	Canada, 1995^22^	pD_ASR	N/A	N/A
Probability of death due to disease at time of first symptomatic relapse	Estimate	pD_Ds1	0.001 (0–0.002)	Tumour will have some degree of resistance to chemotherapy (reflected in low response rates)
Probability of death due to disease at time of second relapse	Estimate	pD_Ds2	0.01 (0–0.02)	Assume tumour will have greater resistance to chemotherapy
Probability of death due to progressive disease	Estimate	pD_ProgDx	0.1 (0–0.2)	Progressing on second-line treatment leads to third line chemotherapy or death
Probability of recurrence, given remission following second line chemotherapy	Estimate	pRecur_OKPostTx2	0.15 (0.05–0.25)	All patients will eventually recur following second-line chemotherapy
Probability of death due to second-line chemotherapy	Estimate	pD_Chemo2	0.001 (0–0.002)	Assume chemotherapy for symptomatic recurrenceis platinum, topotecan, or doxil
Probability of death due to third-line chemotherapy	Estimate	pD_Chemo3	0.005 (0–0.01)	Assume third-line agent is platinum, topotecan, or doxil, but with cumulative toxicity and risk
Probability of disease progression on second-line chemotherapy	Estimate	pProg_Tx2	0.40 (0.20–0.60)	Response rates to second line chemotherapy is approximately 20%–25%
Probability of recurrence, given remission after first-line therapy	Ozols *et al.*, 2001 23; Eltabbakh and Awtrey, 2001 24	pRecur	0.25 (0.15–0.35)	Probability based on 5-year survival rate of 25% (range: 15%–35%); assume RR constant over time
Probability of symptomatic recurrence following remission after first-line therapy	Bast *et al.*, 1983 25	pSymp	0.20 (0.10–0.30)	Among ovarian cancers, 80% express CA-125; patients with CA-125 expression will have an asymptomatic recurrence; remainder (0.2) will be symptomatic
Probability of a positive CA-125 test	Bast *et al.*, 1983 25	pPosTest	0.82 Actual data followβ distribution	Of 101 patients with surgically proven ovarian cancer, 83 are CA-125–positive
Relative risk of death from unrecognized and untreated relapse	Estimate	RR_Death Asymptomatic	*u=*1.00 (0.91–1.1)	Value reflects a hypothetical probability of death from an asymptomatic relapse

**TABLE II:** Incremental results for non-intensive versus intensive monitoring

Summary statistics	Life expectancy	Difference in	
		Quality-adjusted life expectancy	Costs
Expected value (mean)	0.00	−0.28	$801.63
Range	0.00 to 0.01	−0.09, −1.05	$341.00, $3619.00
95% CI	0.00 to 0.01	−0.13 to −0.69	$402.00 to $2099.00

ci = confidence interval.
